# Effects of Side Chain and Peptide Bond Modifications on the Targeting Properties of Stabilized Minigastrin Analogs

**DOI:** 10.3390/ph16020278

**Published:** 2023-02-13

**Authors:** Taraneh Sadat Zavvar, Anton Amadeus Hörmann, Maximilian Klingler, Dominik Summer, Christine Rangger, Laurence Desrues, Hélène Castel, Pierrick Gandolfo, Elisabeth von Guggenberg

**Affiliations:** 1 Department of Nuclear Medicine, Medical University of Innsbruck, 6020 Innsbruck, Austria; 2 Inserm U1245, University Rouen Normandie, 76000 Rouen, France; 3 Institute of Research and Biomedical Innovation (IRIB), 76000 Rouen, France

**Keywords:** minigastrin, cholecystokinin-2 receptor, metabolic stabilization, molecular imaging, radiometals, theranostics, medullary thyroid carcinoma

## Abstract

Different attempts have been made in the past two decades to develop radiolabeled peptide conjugates with enhanced pharmacokinetic properties in order to improve the application for tumor imaging and peptide receptor radionuclide therapy (PRRT), which targets the cholecystokinin-2 receptor (CCK2R). In this paper, the influence of different side chain and peptide bond modifications has been explored for the minigastrin analog DOTA-DGlu-Ala-Tyr-Gly-Trp-(*N*-Me)Nle-Asp-1Nal-NH_2_ (DOTA-MGS5). Based on this lead structure, five new derivatives were synthesized for radiolabeling with trivalent radiometals. Different chemical and biological properties of the new derivatives were analyzed. Receptor interaction of the peptide derivatives and cell internalization of the radiolabeled peptides were studied in A431-CCK2R cells. The stability of the radiolabeled peptides in vivo was investigated using BALB/c mice. Tumor targeting of all ^111^In-labeled peptide conjugates, and of a selected compound radiolabeled with gallium-68 and lutetium-177, was evaluated in BALB/c nude mice xenografted with A431-CCK2R and A431-mock cells. All ^111^In-labeled conjugates, except [^111^In]In-DOTA-[Phe^8^]MGS5, showed a high resistance against enzymatic degradation. A high receptor affinity with IC_50_ values in the low nanomolar range was confirmed for most of the peptide derivatives. The specific cell internalization over time was 35.3–47.3% for all radiopeptides 4 h after incubation. Only [^111^In]In-DOTA-MGS5[NHCH_3_] exhibited a lower cell internalization of 6.6 ± 2.8%. An overall improved resistance against enzymatic degradation was confirmed in vivo. Of the radiopeptides studied, [^111^In]In-DOTA-[(*N*-Me)1Nal^8^]MGS5 showed the most promising targeting properties, with significantly increased accumulation of radioactivity in A431-CCK2R xenografts (48.1 ± 9.2% IA/g) and reduced accumulation of radioactivity in stomach (4.2 ± 0.5% IA/g). However, in comparison with DOTA-MGS5, a higher influence on the targeting properties was observed for the change of radiometal, resulting in a tumor uptake of 15.67 ± 2.21% IA/g for [^68^Ga]Ga-DOTA-[(*N*-Me)1Nal^8^]MGS5 and 35.13 ± 6.32% IA/g for [^177^Lu]Lu-DOTA-[(*N*-Me)1Nal^8^]MGS5.

## 1. Introduction

**Figure 1:**
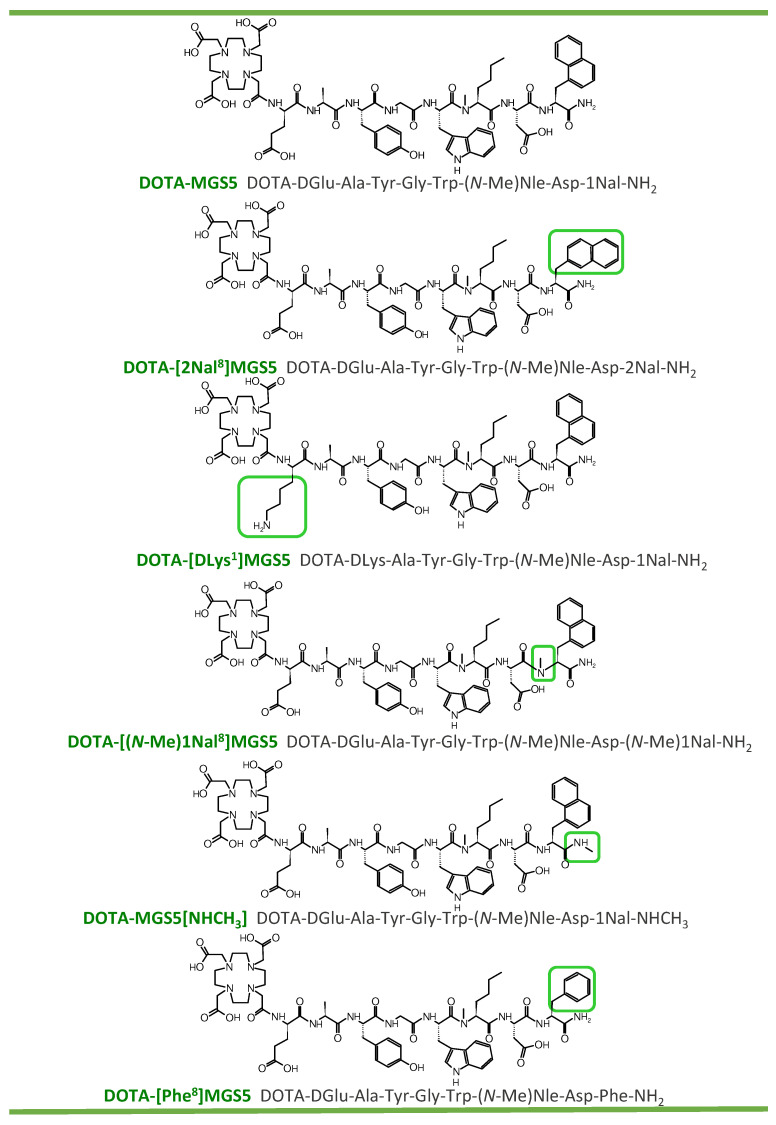
Structural formulas and amino acid sequences of DOTA-MGS5 and the five investigated peptide analogs.

One of the most promising group of targets for both detection and treatment of different types of cancer are G-protein-coupled receptors overexpressed on the tumor cell surface [1]. The cholecystokinin-2 receptor (CCK2R) belongs to this group of targets and is overexpressed in different types of neoplasms, such as medullary thyroid carcinoma (MTC) and small cell lung cancer [2,3]. MTC is a neoplasm with low incidence but connected with limitations in sensitivity and specificity of diagnostic imaging procedures, as well as lack of effective treatment strategies [4,5]. The main treatment strategy at initial diagnosis is surgical intervention consisting of total thyroidectomy with lymphadenectomy. At an advanced stage of the disease, systemic therapy with tyrosine kinase inhibitors may be applied in patients with progressive or symptomatic disease, whereas chemotherapy and external beam radiation therapy play a minor role in the clinical management of MTC [6,7]. Radiolabeled somatostatin analogs, which are very powerful in the clinical management of neuroendocrine tumors, have shown limited success in the diagnosis and therapy of MTC [8]. In the past two decades, different research groups have attempted to develop radiolabeled peptide conjugates targeting CCK2R for tumor receptor imaging and peptide receptor radionuclide therapy (PRRT) [9,10,11]. The first peptide derivatives were derived from the natural ligands cholecystokinin (CCK) and minigastrin (MG). Different modifications were applied to these natural ligands to enhance the pharmacokinetic properties as well as stability. The modifications were applied aiming to overcome limitations such as unfavorable tumor-to-kidney ratio or fast enzymatic degradation, reducing the applicability for imaging and therapy [10,12]. Gastrin analogs with superior CCK2R targeting display a high kidney uptake. Depletion of the penta-Glu sequence within the linear amino acid sequence of MG successfully reduced renal accumulation. However, this modification was associated with shorter physiologic half-life of the peptide, leading to poor diagnostic accuracy [13]. Uptake in non-target organs such as the stomach, with high physiological receptor expression, and the kidney, due to renal elimination, leads to high background activity in non-target tissue and side effects such as nephrotoxicity in PRRT [14,15]. New radiolabeled peptide conjugates have been developed in recent years showing improved targeting properties and have entered clinical studies [16,17,18]. Especially with the new peptide derivative DOTA-MGS5 (DOTA-DGlu-Ala-Tyr-Gly-Trp-(*N*-Me)Nle-Asp-1Nal-NH_2_), higher tumor accumulation and enhanced tumor-to-organ ratios were achieved. Two site-specific substitutions in the C-terminal receptor binding sequence enabled this significant improvement [19]. The first clinical translation of high sensitivity PET imaging with ^68^Ga-labeled DOTA-MGS5 was pursued recently [20]. The main reason for the high tumor uptake of DOTA-MGS5 labeled with different radiometals was found to be a considerable improvement against enzymatic degradation in vivo. In order to study this phenomenon in more detail, starting from the structure of DOTA-MGS5, we have synthesized additional peptide derivatives with varying alternative stabilizing modifications. The impact of these modifications on the receptor affinity, cell internalization, and stability against enzymatic degradation was studied. For selected peptide analogs, the biodistribution profile in the mouse tumor xenograft model was evaluated. As an alternative to substitution of phenylalanine (Phe) with the bulky amino acid 1-naphthylalanine (1Nal) [21], substitution with 2-naphthylalanine (2Nal) was investigated. In addition to *N*-methylated 1-naphthylalanine ((*N*-Me)1Nal) in replacement for oxidation-sensitive methionine (Met), additional *N*-methylation of the peptide bond Asp-1Nal or methylation of the amidated C-terminus was applied to stabilize the peptide backbone. Furthermore, DGlu was replaced with DLys in order to study the influence of charge. The introduction of the uncharged amino acid proline in different positions of the N-terminal amino acid sequence was studied previously [22]. The main aim was to evaluate whether the additional modifications further improve the resistance against enzymatic breakdown. A total of five new radiolabeled MG analogs were studied. The amino acid sequence and structural formula of each peptide are shown in Figure 1. The ^111^In-labeled peptide derivatives have been characterized in vitro and in vivo, allowing for a better comparison with previous results generated by our group. The peptide derivative with the most promising targeting properties was additionally studied when labeled with gallium-68 for potential application for PET imaging, and lutetium-177 for PRRT.

## 2. Results

### 2.1. Peptide Synthesis and Radiolabeling

The investigated DOTA-conjugated MG analogs were synthesized in moderate yields of ≤20% by standard solid-phase peptide synthesis. After purification and characterization by analytical HPLC and MALDI-TOF mass spectrometry (see Table 1), the peptide derivatives were lyophilized in aliquots of 0.5–1 mg and kept at −20 °C. The peptides were dissolved in 20–50% ethanol or phosphate buffered saline (PBS) for further experimental use. Using the standardized radiolabeling protocols described in the method section, the ^111^In-labeled peptides were prepared at an apparent molar activity of 3–7 GBq/µmol and with high radiochemical purity (RCP ≥90%), allowing their use without purification in in vitro tests. Higher apparent molar activities were achieved for the biodistribution studies (indium-111: ~10 GBq/µmole; gallium-68/lutetium-177: ~25 GBq/µmole). To avoid the presence of radioactive impurities in the solutions used in biodistribution studies, the radiolabeling mixtures were subjected to solid phase extraction (SPE) as described before [23]. UV-traces and MALDI-TOF mass spectra of the peptide derivatives, as well as radiochromatograms after labeling with different radiometals, are shown in Figures S1–S3 in the Supplementary Materials.

### 2.2. In Vitro Characterization

The LogD evaluated for the ^111^In-labeled peptides in octanol/PBS resulted in a hydrophilicity ranking of [^111^In]In-DOTA-[Phe^8^]MGS5 (−3.19 ± 0.06) > [^111^In]In-DOTA-[(*N*-Me)1Nal^8^]MGS5 (−2.35 ± 0.27) > [^111^In]In-DOTA-MGS5[NHCH_3_] (−2.33 ± 0.23) > [^111^In]In-DOTA-MGS5 (−2.0 ± 0.1) [19] > [^111^In]In-DOTA-[2Nal^8^]MGS5 (−1.71 ± 0.19) > [^111^In]In-DOTA-[DLys^1^]MGS5 (−1.31 ± 0.11). The introduction of the different amino acid modifications in DOTA-MGS5 only showed a minor influence on its binding to serum proteins. For the time point of 24 h after incubation, lowest values were found for [^111^In]In-DOTA-[Phe^8^]MGS5 (20.2 ± 1.6%), followed by [^111^In]In-DOTA-[DLys^1^]MGS5 (27.8 ± 0.4%), [^111^In]In-DOTA-[2Nal^8^]MGS5 (34.6 ± 0.3%), [^111^In]In-DOTA-[(*N*-Me)1Nal^8^]MGS5 (39.8 ± 4.2%), [^111^In]In-DOTA-MGS5[NHCH_3_] (40.9 ± 3.1%) and [^111^In]In-DOTA-MGS5 (44.3 ± 0.3%) [19]. Similar values were found for most of the radiopeptides at 1 and 4 h after incubation, except for [^111^In]In-DOTA-[Phe^8^]MGS5 for which lower values of 8.1 ± 0.3% and 11.8 ± 2.4% were found, respectively. Incubation of the ^111^In-labeled peptides in fresh human serum for up to 24 h confirmed a high stability in vitro for most of the radioligands investigated. Only for [^111^In]In-DOTA-[Phe^8^]MGS5 the percentage of intact radiopeptide in human serum dropped from 99.5 ± 0.7% 1 h after incubation to 92.5 ± 0.7% and 54.0 ± 0.8% at 4 h and 24 h, respectively. For [^111^In]In-DOTA-[2Nal^8^]MGS5 >90% intact radiopeptide was observed in human serum after 24 h incubation. All other radiopeptides showed >95% intact radiopeptide for the same time point. Given the lowest stability found for [^111^In]In-DOTA-[Phe^8^]MGS5, only minor additional tests were performed for this peptide. The different results of the in vitro characterizations of the ^111^In-labeled peptides are summarized in Table 1 and in Table S1 of DOTA-[(*N*-Me)1Nal^8^]MGS5 labeled with different radiometals, which was also studied. A high complex stability of >90% was confirmed in PBS (*n* = 1).

### 2.3. Receptor Affinity and Cell Internalization Studies 

**Figure 2:**
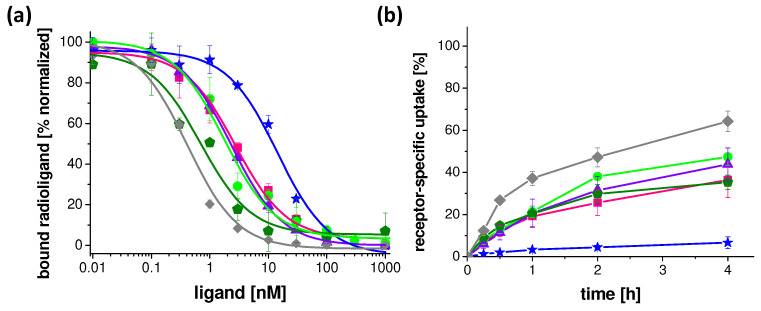
Receptor interaction on A431-CCK2R cells: (**a**) binding curves obtained for the unlabeled peptide derivates from competition assays with [^125^I][3-iodo-Tyr^12^,Leu^15^]gastrin-I; (**b**) Cell internalization of the different ^111^In-labeled peptide derivatives. Gray: previously studied DOTA-MGS5 [19], pink: DOTA-[2Nal^8^]MGS5, green: DOTA-[DLys^1^]MGS5, purple: DOTA-[(N-Me)1Nal^8^]MGS5, blue: DOTA-MGS5[NHCH3], olive: DOTA-[Phe^8^]MGS5.

The IC_50_ values generated from the competitive binding curves obtained with [^125^I][3-iodo-Tyr^12^,Leu^15^]gastrin-I as radioligand and increasing concentrations of the unlabeled peptide derivatives are summarized in Table 2. For all peptides, except DOTA-MGS5[NHCH_3_], IC_50_ values in the low nanomolar (nM) range, comparable to DOTA-MGS5, were found [19]. The receptor affinity of DOTA-MGS5[NHCH_3_] was about ten times lower. Exemplary normalized IC_50_ binding curves for all peptide derivatives are shown in Figure 2. In addition, the EC_50_ values obtained from calcium mobilization assays are shown in Table 2. DOTA-MGS5, DOTA-[2Nal^8^]MGS5 and DOTA-[Phe^8^]MGS5 provoked a similar intracellular Ca^2+^-mobilization, with EC_50_ values in the low nanomolar range, whereas the potency of DOTA-[DLys^1^]MGS5, DOTA-[(*N*-Me)1Nal^8^]MGS5 and DOTA-MGS5[NHCH_3_] was about five times lower.

The receptor-specific internalization was investigated at five different time points from 15 min to 4 h after incubation. All ^111^In-labeled peptides, except [^111^In]In-DOTA-MGS5[NHCH_3_], demonstrated a high receptor-mediated cell internalization with values in the order of 47.2 ± 4.5% for [^111^In]In-DOTA-MGS5, >38.0 ± 1.6% for [^111^In]In-DOTA-[DLys^1^]MGS5, >31.5 ± 6.7% for [^111^In]In-DOTA-[(*N*-Me)1Nal^8^]MGS5, >25.6 ± 6.1% for [^111^In]In-DOTA-[2Nal^8^]MGS5, and >29.7 ± 4.5% for [^111^In]In-DOTA-[Phe^8^]MGS5 at 2 h after incubation [19]. A much lower uptake of 4.4 ± 0.9% was found for [^111^In]In-DOTA-MGS5[NHCH_3_] for the same time point. The cell internalization of each radiopeptide over time is shown in Figure 2. In addition, the cell uptake of DOTA-[(*N*-Me)1Nal^8^]MGS5 labeled with different radiometals is shown in the Supplementary Materials (Figure S4).

### 2.4. Metabolic Studies in BALB/c Mice 

**Figure 3:**
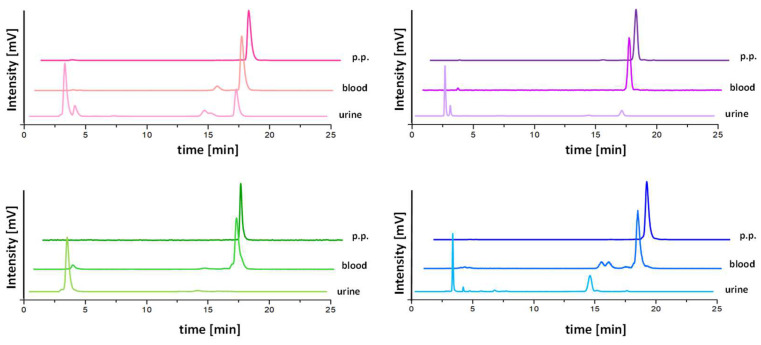
Representative radiochromatograms of the ^111^In-labeled peptides post preparation (p.p.), as well as blood and urine samples obtained from BALB/c mice 10 min p.i. Pink: DOTA-[2Nal^8^]MGS5, green: DOTA-[DLys^1^]MGS5, purple: DOTA-[(*N*-Me)1Nal^8^]MGS5, blue: DOTA-MGS5[NHCH_3_].

In vivo stability studies were performed only for the ^111^In-labeled peptides with confirmed high stability in human serum. After intravenous injection of the radioligands in BALB/c mice, an overall improved resistance against enzymatic degradation could be confirmed for [^111^In]In-DOTA-[2Nal^8^]MGS5, [^111^In]In-DOTA-[DLys^1^]MGS5, [^111^In]In-DOTA-[(*N*-Me)1Nal^8^]MGS5 and [^111^In]In-DOTA-MGS5[NHCH_3_] for the studied time point of 10 min post injection (p.i.). The highest stability in terms of percentage of intact radiopeptide in blood was found for [^111^In]In-DOTA-[(*N*-Me)1Nal^8^]MGS5 (98.4 ± 0.1%). Somewhat lower, but comparable levels were observed for [^111^In]In-DOTA-[DLys^1^]MGS5 (93.9 ± 1.2%). [^111^In]In-DOTA-[2Nal^8^]MGS5 (88.4 ± 0.4%) and [^111^In]In-DOTA-MGS5[NHCH_3_] (71.7 ± 6.2%) showed a further decrease in stability which was comparable to the level of 82.7 ± 3.3% observed for [^111^In]In-DOTA-MGS5 in previous studies [19]. [^111^In]In-DOTA-[(*N*-Me)1Nal^8^]MGS5 showed the highest levels of intact radiopeptide in liver and kidney with values of 94.9 ± 2.8% and 40.2 ± 4.1%, respectively, whereas a higher degree of degradation was found in the urine, resulting in much lower values of intact radiopeptide (15.9 ± 3.4%). The presence of radiometabolites with higher retention time in blood vs. urine confirms the stabilization against metabolic degradation during circulation. In Table 3, the percentage of intact radiopeptide found in all samples examined is summarized for all ^111^In-labeled peptide derivatives. Exemplary radiochromatograms of the radiolabeled peptides, as well as of the analyzed samples obtained from the mice 10 min p.i., are depicted for blood and urine in Figure 3 and for liver and kidney in Figure S5 in the Supplementary Materials.

### 2.5. Biodistribution Studies in BALB/c Nude Mice Xenografted with A431-CCK2R and A431-Mock Cells

**Figure 4:**
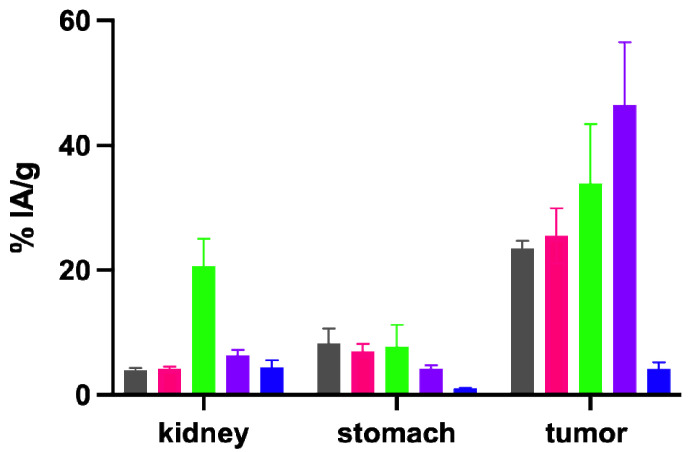
Uptake in kidney, stomach and A431-CCK2R xenografts at 4 h p.i. of the ^111^In-labeled peptides in comparison with [^111^In]In-DOTA-MGS5 [19]. Gray: DOTA-MGS5, pink: DOTA-[2Nal^8^]MGS5, green: DOTA-[DLys^1^]MGS5, purple: DOTA-[(*N*-Me)1Nal^8^]MGS5, blue: DOTA-MGS5[NHCH_3_].

The results of the biodistribution studies performed with the ^111^In-labeled peptides in A431-CCK2R/A431-mock xenografted female athymic BALB/c nude mice for the time point of 4 h p.i. are shown in Table 4. The different radiopeptides investigated were injected in the animals using a constant total peptide amount of 20 pmol. All radiopeptides investigated showed rapid renal excretion, leading to low accumulation in the majority of tissues. For A431-CCK2R xenografts (tumor weight: 0.22 ± 0.14 g, *n* = 24), the highest uptake was observed with [^111^In]In-DOTA-[(*N*-Me)1Nal^8^]MGS5 (48.10 ± 9.15% IA/g). This was significantly increased compared to the value of 23.49 ± 1.25% IA/g (*p* ≤ 0.002) observed for the reference compound [^111^In]In-DOTA-MGS5 in previous studies [19]. [^111^In]In-DOTA-[DLys^1^]MGS5 (33.94 ± 9.53% IA/g) only showed a minor increase in tumor uptake, whereas the uptake value of [^111^In]In-DOTA-[2Nal^8^]MGS5 (25.45 ± 4.45% IA/g) was comparable to [^111^In]In-DOTA-MGS5. In terms of tumor-to-kidney ratio, [^111^In]In-DOTA-[(*N*-Me)1Nal^8^]MGS5 (7.55 ± 0.48) outperformed all other radiopeptides, despite the lower renal uptake observed for [^111^In]In-DOTA-[2Nal^8^]MGS5 (4.21 ± 0.29% IA/g) vs. [^111^In]In-DOTA-[(*N*-Me)1Nal^8^]MGS5 (6.33 ± 0.89%). The high kidney uptake of [^111^In]In-DOTA-[DLys^1^]MGS5 (20.67 ± 4.37% IA/g) and the low tumor uptake of [^111^In]In-DOTA-MGS5[NHCH_3_] (4.16 ± 1.01% IA/g) resulted in an unfavorable tumor-to-kidney ratio of <2. Interestingly, [^111^In]In-DOTA-[(*N*-Me)1Nal^8^]MGS5 showed a clearly reduced accumulation of radioactivity in CCK2R-positive stomach (4.20 ± 0.51% IA/g) as compared to [^111^In]In-DOTA-[2Nal^8^]MGS5 (7.03 ± 1.10) as well as [^111^In]In-DOTA-MGS5 (8.24 ± 2.40%) [19], resulting in the best tumor-to-stomach ratio (11.46 ± 1.82). The uptake in A431-CCK2R xenografts, kidney and stomach is shown for all radiopeptides in Figure 4.

**Figure 5:**
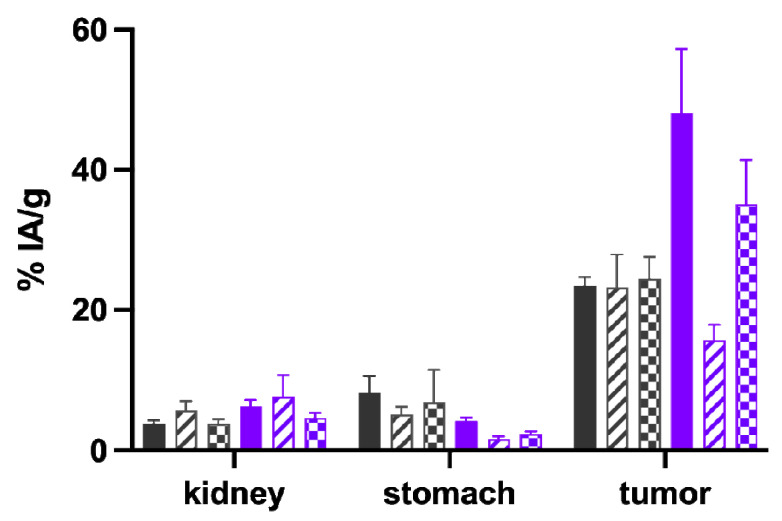
Uptake in kidney, stomach and A431-CCK2R xenografts of radiolabeled DOTA-[(*N*-Me)1Nal^8^]MGS5 in comparison with DOTA-MGS5 [19] at different time points (indium-111 and lutetium-177: 4 h p.i., gallium-68: 1 h p.i.). Gray: [^111^In]In-DOTA-MGS5, striped gray: [^68^Ga]Ga-DOTA-MGS5, checked gray: [^177^Lu]Lu-DOTA-MGS5, purple: [^111^In]In-DOTA-[(*N*-Me)1Nal^8^]MGS5, striped purple: [^68^Ga]Ga-DOTA-[(*N*-Me)1Nal^8^]MGS5, checked purple: [^177^Lu]Lu-DOTA-[(*N*-Me)1Nal^8^]MGS5.

The most promising biodistribution results were obtained for ^111^In-labeled DOTA-[(*N*-Me)1Nal^8^]MGS5. Using this peptide, additional studies were performed for the radiometal conjugates with gallium-68 and lutetium-177 (see Figure 5 and Table 5) and compared to previously studied DOTA-MGS5 [19,24]. Even though a constant total peptide amount of 20 pmol was injected for all radiopeptides studied, [^68^Ga]Ga-DOTA-[(*N*-Me)1Nal^8^]MGS5 showed a significantly lower uptake in CCK2R-positive tumor xenografts (15.67 ± 2.21% IA/g vs. 23.25 ± 4.70% IA/g for [^68^Ga]Ga-DOTA-MGS5, *p* ≤ 0.05). [^177^Lu]Lu-DOTA-[(*N*-Me)1Nal^8^]MGS5, similarly to [^111^In]In-DOTA-[(*N*-Me)1Nal^8^]MGS5, showed a significantly improved tumor accumulation (35.13 ± 6.32% vs. 22.89 ± 4.67% for [^177^Lu]Lu-DOTA-MGS5, *p* ≤ 0.05). The stomach uptake remained at very low levels, but was significantly reduced only for gallium-68 (gallium-68: 5.12 ± 1.13% vs. 1.63 ± 0.41%, *p*≤ 0.01; lutetium-177: 2.37 ± 0.33 vs. 6.26 ± 4.28%, *p* ≥ 0.05). For gallium-68, the tumor-to-kidney ratio was in the favor of DOTA-MGS5 vs. DOTA-[(*N*-Me)1Nal^8^]MGS5 (*p* ≤ 0.05), and only slightly improved for [^177^Lu]Lu-DOTA-[(*N*-Me)1Nal^8^]MGS5 vs. [^177^Lu]Lu-DOTA-MGS5 (*p*≥ 0.05). 

The non-specific uptake in A431-mock xenografts (tumor weight: 0.18 ± 0.13 g, *n* = 24) was below 1% IA/g for all peptide derivatives labeled with indium-111 and lutetium-177, and slightly increased for gallium-68 (1.51 ± 0.98% IA/g). 

## 3. Discussion

CCK2R targeting in patients with MTC and other malignancies characterized by CCK2R overexpression using radiolabeled peptide analogs derived from endogenous ligands has high potential for diagnostic and therapeutic application [10,25]. However, unfavorable properties such as excessive renal retention or poor enzymatic stability have led to limitations in the clinical applicability [13,25].

Various modifications to the peptide sequence have been investigated in order to stabilize the peptide derivatives against degradation in vivo and enhance the tumor targeting properties. Rapid metabolization in vivo with major cleavage sites within the C-terminal receptor specific part of the peptide sequence was reported for different CCK2R targeting peptide analogs [26]. In recent studies by our group, it was shown that by applying site-specific modifications within the C-terminal receptor-specific sequence, it is possible to prevent enzymatic degradation. This stabilization also resulted in improved tumor uptake [19,21,27]. In the peptide derivative DOTA-MGS5, two substitutions were introduced in the receptor-specific sequence “Trp-Met-Asp-Phe-NH_2_”. Oxidation-sensitive Met was replaced by (*N*-Me)Nle, and Phe was replace by 1Nal. The combination of these two substitutions enabled a considerable improvement of the targeting properties. DOTA-MGS5 radiolabeled with gallium-68 is currently being studied in a first clinical trial [19]. In this study, further modifications were applied to the amino acid sequence of DOTA-MGS5 and the effects of these modifications on the pharmacokinetic properties and the tumor targeting properties were evaluated. The introduction of 2Nal in replacement of 1Nal, as well as additional *N*-methylation of the peptide bond at Asp-Nal and of the amidated C-terminus was investigated. Furthermore, replacement of DGlu in position 1 by DLys was investigated. 

The in vitro characterization confirmed that the ^111^In-labeled peptide derivatives still show a favorable hydrophilicity and binding to serum proteins comparable to [^111^In]In- DOTA-MGS5 [19]. The introduction of the bulky amino acid 1Nal/2Nal, as well as the *N*-methylation in different parts of the peptide backbone, resulted in somewhat lower hydrophilicity and increased serum protein binding of 28–44%. Only for [^111^In]In-DOTA-[Phe^8^]MGS5, which is missing the introduction of 1Nal, was a higher hydrophilicity and lower protein binding of 20% found. Increased serum protein binding in addition to a possible prolonged blood circulation, was associated with an improved stability of the radiopeptides in human serum. All compounds, except [^111^In]In-DOTA-[Phe^8^]MGS5, exhibited a high stability in human serum. *N*-methylation at the C-terminus led to a reduced receptor affinity of DOTA-MGS5[NHCH_3_]. All other peptide conjugates showed a receptor affinity in the low nanomolar range, comparable to DOTA-MGS5 [19]. Interestingly, for three peptide conjugates, namely DOTA-[DLys^1^]MGS5, DOTA-[(*N*-Me)1Nal^8^]MGS5 and DOTA-MGS5[NHCH_3_], higher EC_50_ values were found, suggesting a possible decreased activity. However, all peptide derivatives showed a similar maximum response of Ca^2+^ release. [^111^In]In-DOTA-[Phe^8^]MGS5, with a single substitution with (*N*-Me)Nle within the C-terminal receptor specific region, showed a receptor-specific cell internalization of 29.7 ± 4.5% after 2 h incubation. We have previously reported on [^111^In]In-DOTA-MGS1 with a single substitution with 1Nal for which a cell internalization of 26.2 ± 3.4% was found. [^111^In]In-DOTA-MGS1, comparably to [^111^In]In-DOTA-[Phe^8^]MGS5, showed a low stability of ~50% in human serum which was associated with low unfavorable tumor targeting of 1.23 ± 0.15% IA/g 4 h p.i. [21]. Due to this low stability against enzymatic degradation, no further in vivo assessments were performed for [^111^In]In-DOTA-[Phe^8^]MGS5. Combined substitution with (*N*-Me)Nle and 1Nal is necessary for efficient stabilization against enzymatic degradation. The different spatial orientation of the naphthyl side chain of 1Nal and 2Nal had no significant impact in terms of affinity, stability and cell uptake. The same holds true for the introduction of DLys in replacement of DGlu.

Metabolic studies in BALB/c mice were performed to confirm a high stability against enzymatic breakdown of the ^111^In-labeled peptide analogs in vivo. Altogether, the results were in line with the in vitro stability studies in human serum, but allowed for a more accurate assessment of the stability against enzymatic breakdown in vivo. For the studied time point of 10 min after injection, the stability of [^111^In]In-DOTA-[2Nal^8^]MGS5 and [^111^In]In-DOTA-[DLys^1^]MGS5 was similar to [^111^In]In-DOTA-MGS5 [19], whereas the stability of [^111^In]In-DOTA-MGS5[NHCH_3_] was slightly lower. The most stable radiopeptide of this series studied was [^111^In]In-DOTA-[(*N*-Me)1Nal^8^]MGS5, for which almost no degradation could be observed. *N*-methylation of the peptide bond between Asp and 1Nal seemed most promising in achieving a further improvement in tumor targeting. In line with these results, [^111^In]In-DOTA-[(*N*-Me)1Nal^8^]MGS5 showed the highest improvement in tumor uptake in the A431-CCK2R xenografts (48.10 ± 9.15% IA/g at 4 h p.i.). [^111^In]In-DOTA-[2Nal^8^]MGS5 showed a very similar biodistribution profile when compared to [^111^In]In-DOTA-MGS5, confirming that the different spatial orientation of the naphthyl group has no influence on the receptor interaction. Substitution of DGlu with DLys in [^111^In]In-DOTA-[DLys^1^]MGS5 also resulted in improved tumor accumulation. However, the introduction of the positive charge also led to considerably higher kidney uptake (~20% IA/g), reducing the tumor-to-kidney ratio. A much lower tumor accumulation of 4.16 ± 1.01% IA/g at 4 h p.i. was found for [^111^In]In-DOTA-MGS5[NHCH_3_] with C-terminal *N*-methylation. Still, the tumor uptake was almost two times higher when compared to the peptide conjugate without site-specific amino acid substitutions within the C-terminal receptor-specific region. A tumor uptake of 2.49 ± 0.92% IA/g was previously reported for ^111^In-labled DOTA-MG11 (DOTA-DGlu-Ala-Tyr-Gly-Trp-Met-Asp-Phe-NH_2_) in A431-CCK2 xenografts at 4 h p.i. using an injected peptide amount of 10 pmol [28]. The results show that C-terminal *N*-methylation allows for improved stabilization against metabolic degradation. Despite reduced receptor affinity, receptor-specific tumor targeting can still be achieved in vivo. Previous data reported by our group confirmed complete loss of receptor affinity when replacing the C-terminal amide with a free carboxylic group [23]. [^111^In]In-DOTA-[(*N*-Me)1Nal^8^]MGS5 also showed a superior tumor-to-kidney, and especially tumor-to-stomach, ratio compared to [^111^In]In-DOTA-MGS5. For this reason, this conjugate was further evaluated with alternative radiometals. Interestingly, a considerable influence of the radiometal on the tumor targeting profile was found for DOTA-[(*N*-Me)1Nal^8^]MGS5, whereas for DOTA-MGS5 no significant influence was observed. DOTA-[(*N*-Me)1Nal^8^]MGS5 radiolabeled with different radiometals generally showed a somewhat higher kidney uptake, but considerably reduced stomach uptake when compared to the radiolabeled DOTA-MGS5 counterparts. Non-specific uptake in most of the tissues was also slightly increased. DOTA-MGS5, on the other side, showed a constant tumor uptake of ~25% IA/g independently of the radiometal. For [^68^Ga]Ga-DOTA-[(*N*-Me)1Nal^8^]MGS5, the uptake in A431-CCK2R xenografts was reduced (15.67 ± 2.21% IA/g at 1 h p.i.), whereas for [^177^Lu]Lu-DOTA-[(*N*-Me)1Nal^8^]MGS5 an improvement in tumor targeting (35.13 ± 6.32% IA/g at 4 h p.i.) was found. We believe that differences in receptor affinity, rather than pharmacokinetic properties, may explain this finding. In additional studies performed for DOTA-[(*N*-Me)1Nal^8^]MGS5 labeled with different radiometals, similar serum protein binding and stability for incubation in human serum were observed. Cell uptake in A431-CCK2R cells, similarly to the varying tumor uptake, was higher for [^111^In]In-DOTA-[(*N*-Me)1Nal^8^]MGS5 and [^177^Lu]Lu-DOTA-[(*N*-Me)1Nal^8^]MGS5, as compared to [^68^Ga]Ga-DOTA-[(*N*-Me)1Nal^8^]MGS5. However, no competitive binding assays for DOTA-[(N-Me)NaI^8^]MGS5 complexed with natural metals were performed within this work to investigate possible differences in receptor affintiy. A peptide conjugate with consistent properties independent of the radiometal may be preferable for clinical application. Still, the tumor uptake of the new peptide derivative is considerably improved. A much lower tumor uptake of 6–9% IA/g was previously reported in A431-CCK2R xenografts for [^111^In]In-CP04 (PP-F11, MG48) and [^177^Lu]Lu-PP-F11N at 4 h p.i. when using an injected peptide amount of 10–30 pmol [29,30,31].

Altogether, our results confirm that enhancing the in vivo stability of radiolabeled minigastrin analogs by site-specific substitutions within the C-terminal receptor-specific region has a beneficial effect on tumor targeting properties. When comparing our results with other radiolabeled MG analogs which are currently under clinical investigation, DOTA-MGS5 and DOTA-[(*N*-Me)1Nal^8^]MGS5 exhibit a two to five-fold increase in tumor uptake. So far, only the bifunctional chelator DOTA has been used for radiolabeling with different trivalent radiometals. In our next studies, we will investigate the influence of different chelators on the biodistribution profile and targeting properties of the new peptide derivatives.

## 4. Materials and Methods

### 4.1. Materials and Analytics

Reagents of analytical grade were obtained from different commercial suppliers. [^111^In]InCl_3_ (Mallinckrodt Medical, Petten, The Netherlands) and non-carrier-added [^177^Lu]LuCl_3_ (Isotope Technologies Garching) were used for radiolabeling. [^68^Ga]GaCl_3_ was obtained from a commercial ^68^Ge/^68^Ga generator with nominal activity of 1850 MBq (Eckert and Ziegler, Berlin, Germany) and eluted with 0.1 N HCl solution (Rotem, Leipzig, Germany).

Radioiodination of human [Leu^15^]gastrin-I (Bachem, Bubendorf, Switzerland) was performed with chloramine-T and carrier-free Na [^125^I]I (PerkinElmer, Boston, MA), as previously described [23]. [^125^I][3-iodo-Tyr^12^,Leu^15^]gastrin-I was separated from [Leu^15^]gastrin-I by HPLC purification and stored in aliquots at −20°C.

All compounds were synthesized using standard solid phase synthesis described previously [23]. The peptide derivatives were purified by HPLC purification and characterized by matrix-assisted laser desorption ionization time-of-flight (MALDI-TOF) mass spectrometry (Bruker microflex^®®^, Bruker Daltonics, Bremen, Germany) and analytical HPLC.

A Gilson 322 chromatography system (Gilson International, Limburg, Germany) equipped with Gilson UV/VIS-155 multiwavelength detector and a Eurosil Bioselect Vertex Plus C18A precolumn (300 Å, 5 μm, 30 cm × 8 mm), as well as a Eurosil Bioselect Vertex Plus C18A column (300 Å, 5 μm, 3 cm × 8 mm) (Knauer, Berlin, Germany), was used for preparative HPLC purification. An HPLC gradient with a flow rate of 2 mL/min was applied starting from 80% solvent A (water containing 0.1% trifluoroacetic acid (TFA)) and increasing concentrations of solvent B (acetonitrile (ACN) containing 0.1% TFA): 0−4 min 20% B, 4−24 min 20−60% B, 24−26 min 60% B, 26−27 min 60− 80% B, 27−28 min 80% B, 28−29 min 80−20% B, 29−37 min 20% B.

For analytical HPLC analysis, an UltiMate 3000 chromatography system (Dionex, Germering, Germany) consisting of an HPLC pump, a variable UV detector (UV−vis at λ = 280 nm), an autosampler, and a radiodetector (GabiStar, Raytest, Straubenhardt, Germany), equipped with a Phenomenex Jupiter Proteo C12 column (90 Å, 4 μm, 25 cm × 4.6 mm) (Phenomenex Ltd., Aschaffenburg, Germany) was used. The following gradient system with flow rate set to 1 mL/min was applied: 0−3 min 10% B, 3−18 min 10−55% B, 18−20 min 80% B, 20−21 min 80−10% B, 21−25 min 10% B. The radiodetector allowed us to adjust the sensitivity for the measurement of radioactive samples. Usually, the standard loop of 5 µL was used. Samples with low radioactivity obtained from stability studies were measured with a 250 µL loop, allowing for improved sensitivity when analyzing samples with low radioactivity.

### 4.2. Peptide Synthesis and Radiolabeling

The peptides were synthesized on 50–80 mg Rink Amide MBHA resin with a capacity of 0.5 mmol/g resin (Novabiochem, Hohenbrunn, Germany). The reactive side chains of the amino acids were protected with tert-butyl ester for Asp and DGlu, tert-butyl ether for Tyr, tert-butyloxycarbonyl (BOC) for Trp and 1-(4,4-dimethyl-2,6-dioxocyclohex-1-ylidene)ethyl (Dde) for DLys. The coupling reactions with the Fmoc-protected amino acids (5-fold excess) were performed with O-(7-Azabenzotriazole-1-yl)-*N*,*N*,*N*′,*N*′-tetramethyluronium hexa-fluorophosphate (HATU), 1-hydroxy-7-aza-benzotriazole (HOAt), and *N*-methyl-2-pyrrolidinone (NMP), adjusting the pH to 8–9 with *N*,*N*’-diisopropylethylamine (DIPEA). *N*-methylation of the peptide bond between Asp and 1Nal and of the C-terminal amide in DOTA-MGS5[NHCH_3_] was accomplished following a published protocol [32]. Coupling of DOTA-tris(tert-butyl ester) was performed with a 3-fold molar excess. Cleavage of the peptides from the resin and of the acid-labile protecting groups was carried out using a mixture of trifluoroacetic acid (TFA), triisopropylsilane (TIS), and water (95/2.5/2.5, *v/v/v*). Dde was cleaved using a mixture of 9.6 mL DMF and 400 µL hydrazine (45 min, room temperature). The crude peptides were precipitated and washed with ice-cold diethlyether prior to preparative HPLC purification, MALDI-TOF MS characterization and analytical HPLC.

For labeling with indium-111, 10−20 μg of the DOTA-conjugates were incubated with ~100 μL of [^111^In]InCl_3_ (30−70 MBq, in 0.05 M HCl) and 0.4 M sodium acetate/0.24 M gentisic acid solution adjusted to pH 5 (1.2-fold the volume of [^111^In]InCl_3_) at 95 °C for 20 min. The radiochemical purity was determined using the analytical HPLC system described above. For in vivo stability studies, as well as biodistribution studies, the radiolabeling mixture was subjected to an additional purification step on a C18 or tC18 SepPak^®®^ Light cartridge (Waters, Milford) to remove any free radiolabeled hydrophilic species. The solutions injected in animals were formulated in PBS and <2% ethanol, contained minor amounts of sodium bicarbonate solution for pH adjustment, and a 20-fold molar excess of diethylenetriaminepentaacetic acid (DTPA) to capture released indium-111. For DOTA-[(*N*-Me)1Nal^8^]MGS5, radiolabeling was performed with gallium-68 (10–20 μg, 50−300 MBq) and lutetium-177 (10–20 μg, 50−250 MBq) using previously published protocols [19].

### 4.3. In Vitro Characterization

The distribution coefficient (LogD) in octanol/PBS was determined by mixing 0.5 mL of the radiolabeled DOTA peptides diluted in PBS (100 pmol/mL) and 0.5 mL octanol in a 1.5 mL microcentrifuge tube (*n* = 8). After shaking for 15 min (MS3 Basic, IKA, Staufen, Germany) at 1500 rpm and a waiting time of 10 min to ensure appropriate separation of the two phases, 100 µL aliquots of each phase were pipetted into 4.5 mL tubes. The tubes were measured using a γ-counter (2480 Wizard2 3″, PerkinElmer Life Sciences and Analytical Instruments, formerly Wallac Oy, Turku, Finland) and the LogD value was determined using following equation:

$$LogD=Log\left(\frac{cpm_{octanol\;phase}}{cpm_{PBS\;phase}}\right)$$

Protein binding was determined by incubation of the radiolabeled peptides in human serum at 37 °C (500 pmol/mL, *n* = 2). For the time points of 1, 4, and 24 h after incubation, samples of 25 µL were subjected to Sephadex G-50 size exclusion chromatography (GE Healthcare Illustra, Little Chalfont, UK). The column and the eluate were measured using the γ-counter and the percentage of protein binding was calculated.

In vitro stability studies were performed in human serum (*n* = 2). The radiolabeled peptides (1000 pmol/mL) were incubated at 37 °C and a sample of 100 μL was taken at 1, 4, and 24 h after incubation. After the precipitation of proteins with ACN at 1:1.5 (*v/v*), centrifugation (14,000 rpm, 2 min, centrifuge 5424, Eppendorf AG, Germany) and dilution with water at 1:1 (*v/v*), an aliquot of 100 μL of this solution was analyzed by HPLC. Radioligand breakdown was monitored and the percentage of intact radiopeptide was calculated using Chromeleon Dionex Software (Version 7.2.9.11323). Additionally, the radiolabeled peptides (1000 pmol/mL) were incubated in PBS (*n* = 1) at room temperature to confirm the complex stability as a control.

In vitro characterization was undertaken for DOTA-[(*N*-Me)1Nal^8^]MGS5 radiolabeled with gallium-68 and lutetium-177 (see Table S1 of the Supplementary Materials).

### 4.4. Receptor Affinity and Cell Internalization Studies

A431 human epidermoid carcinoma cells overexpressing human CCK2R through stable transfection (A431-CCK2R) and the same cell line transfected with the empty vector (A431-mock) were used for cell assays. The cells are constantly available in our laboratory and were originally provided by Dr. Luigi Aloj [33]. All cell culture media and reagents were obtained from Invitrogen Corporation (Lofer, Austria) or Sigma-Aldrich (Darmstadt, Germany). Dulbecco’s modified Eagle’s medium (DMEM) supplemented with 10% (*v/v*) fetal bovine serum and 1% of 100x penicillin−streptomycin−glutamine was used for cell culture. The cells were passaged three times per week using a 10x 2.5% trypsin-EDTA solution in a 1:2–1:3 ratio.

Receptor affinity studies of the DOTA-conjugates for the CCK2R was competitively evaluated against [^125^I][3-iodo-Tyr^12^,Leu^15^]gastrin-I. The binding assays were performed in 96-well filter plates (MultiScreenHTS-FB, Merck Group, Darmstadt, Germany) as previously described [19]. A431-CCK2R cells (200,000–400,000/well) were incubated for 1 h at RT with the peptides (concentration range of 0.01–1000 nM) and [^125^I][3-iodo-Tyr^12^,Leu^15^]gastrin-I (~25,000 cpm). The incubation was arrested by filtering the medium by vacuum and rinsing twice rapidly with ice-cold binding assay buffer (10 mM TRIS/139 mM NaCl, pH 7.3). The filters were removed from the plate, transferred into 4.5 mL tubes and measured in the γ-counter. Data were normalized from 0 to 100 for graphical representation of exemplary binding curves. Origin software (Microcal Origin 6.1, Northampton, MA, USA) was used to calculate the half maximal inhibitory concentration (IC_50_) values by nonlinear regression and expressed as mean ± sd for three independent experiments performed in triplicates. An additional cell uptake study at 2 h after incubation was performed for DOTA-[(*N*-Me)1Nal^8^]MGS5 radiolabeled with gallium-68 and lutetium-177 (see Figure S4 of the Supplementary Materials).

Analog-induced receptor activation was evaluated by a calcium mobilization assay using the calcium-sensitive dye fluo-4 AM (ThermoFisher Scientific, Montigny-Le-Bretonneux, France), as previously described [22]. Briefly, A431-CCK2R cells were seeded (60,000/well) in flat clear bottom black 96-well plates (Corning, Bagneaux-sur-Loing, France) coated with poly-L-ornithine (100 µg/mL, 1 h, 37 °C) (Sigma Aldrich, Saint Quentin Fallavier, France). Cytosolic Ca^2+^ variations were measured before and after application of 50 µL of graded concentrations of the investigated peptides in the vicinity of the cells, and the effects on Ca^2+^ mobilization were measured. Data were background corrected and normalized for the amplitudes caused by the medium alone (0%, no self-effect) or 10^−6^ M pentagastrin (100%, maximal Ca^2+^ mobilization response). Prism 4.0 software was used to calculate the EC_50_ values and the results were expressed as mean ± SEM for at least three independent experiments performed in triplicates.

The cell internalization of the radiolabeled peptides was measured on A431-CCK2R and A431-mock cells. The cells (1.0 × 10^6^ cells per well) were seeded in six-well plates and grown for 48 h. On the day of the experiment, the medium was removed and the cells washed twice with ice-cold internalization medium (medium supplemented with 1% (*v*/*v*) fetal bovine serum). The cells were incubated with the ^111^In-labeled peptides in a total volume of 1.5 mL in triplicates at 37 °C (final peptide concentration of 0.4 nM). At defined time points, the medium was removed and the cells were washed with ice-cold internalization medium, followed by treatment with 50 mM glycine buffer pH 2.8, 0.1 M NaCl (2×, 5 min) to remove the membrane-bound radioligand. The cells were separately collected with 1 M NaOH (2 × 1 mL) (internalized radioligand). The collected fractions (supernatant, acid wash, lyzed cells) were measured in the γ-counter together with a standard corresponding to the total radioactivity added to each well. For each time point, the percentage of radioactivity internalized by the cells, in relation to the total radioactivity added, was calculated. The receptor-specific uptake in A431-CCK2R cells was determined by subtraction of the non-specific uptake found in A431-mock cells. For each time point and radiopeptide studied, the mean value and standard deviation for the values obtained from three assays performed in triplicates was calculated.

### 4.5. Metabolic Studies in BALB/c Mice

All animal experiments were approved by the Austrian Ministry of Science (BMWF-66.011/0075- WF/V/3b/2016) and performed in accordance with the Austrian Animal Protection Act. Metabolic studies characterizing the stability of selected radioligands against enzymatic degradation were carried out in 7 to 8 week old female BALB/c mice (Charles River, Sulzfeld, Germany). The mice (two animals for each peptide) were intravenously injected into a lateral tail vein with 5–10 MBq of the ^111^In-labeled peptide derivatives, corresponding to 2 nmol total peptide. At the time point of 10 min p.i. the animals were euthanatized by cervical dislocation. A sample of urine and blood was immediately collected. Liver and kidneys were dissected, mixed with 20 mM HEPES buffer pH 7.3 (1:1, *w*/*v*) and homogenized using an Ultra-Turrax T8 homogenizer (IKA-Werke, Staufen, Germany) for 1 min. All samples taken were analyzed by analytical HPLC using the low-sensitivity loop for urine and the high-sensitivity loop for blood, liver and kidney. Before injection into the HPLC system, all samples except urine were treated with ACN and diluted with water as described for stability studies in human serum. The percentage of intact radiopeptide in the analyzed samples was calculated by integration of the different peaks found in the radiochromatogram using Chromeleon Dionex Software (Version 7.2.9.11323).

### 4.6. Biodistribution Studies in BALB/c Nude Mice Bearing A431-CCK2R/A431-Mock Xenografts

The overall biodistribution and tumor targeting properties of selected radiolabeled peptides were evaluated in female athymic BALB/c nude mice (Charles River, Sulzfeld, Germany, age of 7 to 9 weeks). After tumor induction with 2 × 10^6^ A431-CCK2R or A431-mock cells (subcutaneously injected in 200 μL DMEM into the right and left flank) and a waiting period of 10–14 days (sufficient to achieve tumors of ∼0.25 g), mice were randomly divided into groups of four and treated with a bolus injection of 150 µL of the injectable solution containing the radiolabeled peptide via a lateral tail vein to achieve an injected radioactivity of 150–200 kBq and a total injected peptide amount of ~20 pmol (*n* = 4 for each radiopeptide). For DOTA-[(*N*-Me)1Nal^8^]MGS5, additional biodistribution studies were performed with gallium-68 (~0.5 MBq, 20 pmol) and lutetium-177 (~0.5 MBq, 20 pmol). The animals were euthanatized by cervical dislocation at 1 h (gallium-68) or 4 h p.i. (indium-111/ lutetium-177). The tumors and other tissues (blood, lung, heart, muscle, spleen, intestine, liver, kidney, stomach, and pancreas) were dissected, weighed, and the radioactivity measured in the γ-counter. The rest of the body and a standard of the solution injected was also measured together with the collected samples. The percentage of injected activity per gram of tissue (% IA/g) and the tumor-to-organ activity ratio of selected tissues were calculated for each mouse and time point studied. Origin software (Microcal Origin 6.1, Northampton, MA, USA) was used for statistical analysis (independent t-test for two populations; significance level *p* < 0.05).

## 5. Conclusions

The significant progress achieved in recent years in the development of stabilized radiolabeled MG analogs resulted in new peptide derivatives with enhanced pharmacokinetic profile and improved targeting properties. First clinical studies have been initiated for targeted imaging and therapy of CCK2R positive malignancies. In this study, we have applied different modification strategies with a focus on the C-terminal receptor-specific sequence of the peptide. From the five new peptide derivatives studied, [^111^In]In-DOTA-[(*N*-Me)1Nal^8^]MGS5 showed the most promising targeting properties in terms of high tumor uptake and favorable tumor-to-kidney, as well as tumor-to-stomach, ratio. Further evaluation with different radiometals revealed that the change of the radiometal had a significantly higher impact on the targeting properties of DOTA-[(*N*-Me)1Nal^8^]MGS5 compared to DOTA-MGS5, supporting the ongoing clinical translation of DOTA-MGS5.

## Figures and Tables

**Table 1:** Summary of the analytical data of the peptide derivatives, including previously studied DOTA-MGS5 [19].

Compound	Calculated Mass (*m*/*z* [M+H]^+^)	Found Mass (*m*/*z* [M+H]^+^)	Binding to Serum Proteins (%) * n = 2	LogD *n* = 8	Intact Radiopeptide in Human Serum (%) * n = 2
DOTA-MGS5	1449.7	1449.3	44.3 ± 0.3	−2.0 ± 0.1	97.2 ± 0.6
DOTA-[2Nal^8^]MGS5	1449.7	1447.7	34.6 ± 0.3	−1.7 ± 0.2	92.0 ± 1.2
DOTA-[DLys^1^]MGS5	1448.7	1450.8	27.8 ± 0.4	−1.3 ± 0.1	98.1 ± 1.1
DOTA-[(*N*-Me)1Nal^8^]MGS5	1463.7	1463.7	39.8 ± 4.2	−2.4 ± 0.3	99.6 ± 0.1
DOTA-MGS5[NHCH_3_]	1463.7	1463.8	40.9 ± 3.1	−2.3 ± 0.2	98.1 ± 0.4
DOTA-[Phe^8^]MGS5	1399.7	1399.6	20.2 ± 1.6	−3.2 ± 0.1	54.0 ± 0.8

* as determined for the time point of 24 h after incubation.

**Table 2:** IC_50_ and EC_50_ values of the peptide analogs calculated from competitive binding assays and calcium mobilization assays on A431-CCK2R cells in comparison with previously studied DOTA-MGS5.

Peptide	IC_50_ (nM)	EC_50_ (nM)
DOTA-MGS5	0.4 ± 0.2 [19]	7.9 ± 1.9
DOTA-[2Nal^8^]MGS5	1.7 ± 0.1	12.8 ± 2.4
DOTA-[DLys^1^]MGS5	0.9 ± 0.8	43.5 ± 14.1
DOTA-[(*N*-Me)1Nal^8^]MGS5	2.7 ± 0.3	63.4 ± 19.9
DOTA-MGS5[NHCH_3_]	13.2 ± 1.5	41.0 ± 14.7
DOTA-[Phe^8^]MGS5	0.7 ± 0.1	6.3 ± 3.6

**Table 3:** HPLC analysis of samples obtained from metabolic studies in BALB/c mice analyzing the percentage of intact radiopeptide 10 min p.i. Data of previously studied [^111^In]In-DOTA-MGS5 are added for comparison [19].

	[^111^In]In-DOTA-MGS5	[^111^In]In-DOTA-[2Nal^8^]MGS5	[^111^In]In-DOTA-[DLys^1^]MGS5	[^111^In]In-DOTA- [(*N*-Me)1Nal^8^]MGS5	[^111^In]In-DOTA-MGS5[NHCH_3_]
blood	82.7 ± 3.3	88.4 ± 0.4	93.9 ± 1.2	98.4 ± 0.1	71.7 ± 6.2
liver	87.8 ± 2.1	83.9 ± 0.1	82.5 ± 2.4	94.9 ± 2.8	78.1 ± 3.7
kidney	21.5 ± 1.7	24.7 ± 0.3	20.6 ± 3.8	40.2 ± 4.1	7.4 ± 8.6
urine	11.5 ± 2.5	28.2 ± 1.0	5.8 ± 0.2	15.9 ± 3.4	1.3 ± 0.2

**Table 4:** Biodistribution and selected tumor-to-organ ratios of the ^111^In-labeled peptides in A431-CCK2R/A431-mock xenografted BALB/c nude mice 4 h p.i. All biodistribution values are reported as mean ± SD (*n* = 4). Data of previously studied [^111^In]In-DOTA-MGS5 are added for comparison [19].

%IA/g	[^111^In]In-DOTA-MGS5	[^111^In]In-DOTA-[2Nal^8^]MGS5	[^111^In]In-DOTA-[DLys^1^]MGS5	[^111^In]In-DOTA- [(*N*-Me)1Nal^8^]MGS5	[^111^In]In-DOTA-MGS5[NHCH_3_]
blood	0.05 ± 0.03	0.06 ± 0.003	0.20 ± 0.09 *	0.19 ± 0.07 *	0.08 ± 0.07
lung	0.09 ± 0.05	0.13 ± 0.03	0.30 ± 0.11 *	0.21 ± 0.07 *	0.07 ± 0.01
heart	0.05 ± 0.02	0.08 ± 0.01 *	0.22 ± 0.06 *	0.14 ± 0.03 *	0.06 ± 0.02
muscle	0.26 ± 0.28	0.04 ± 0.01	0.13 ± 0.05	0.12 ± 0.05	0.04 ± 0.02
spleen	0.16 ± 0.04	0.18 ± 0.05	0.54 ± 0.12 *	0.32 ± 0.05 *	0.13 ± 0.02
intestine	1.15 ± 0.31	0.95 ± 0.26	1.96 ± 0.87	0.81 ± 0.10	0.59 ± 0.46
liver	0.46 ± 0.03	0.66 ± 0.07 *	1.94 ± 0.52 *	1.39 ± 0.16 *	0.36 ± 0.11
kidneys	3.88 ± 0.45	4.21 ± 0.29	20.67 ± 4.37 *	6.33 ± 0.89 *	4.42 ± 1.15
pancreas	1.6. ± 0.50	1.84 ± 0.52	2.27 ± 1.20	1.37 ± 0.22	0.40 ± 0.04 *
stomach	8.24 ± 2.40	7.03 ± 1.10	7.73 ± 3.49	4.20 ± 0.51 *	0.96 ± 0.17 *
A431-mock xenograft	0.3 ± 0.12	0.17 ± 0.04	0.56 ± 0.27	0.38 ± 0.08	0.19 ± 0.08
A431-CCK2R xenograft	23.49 ± 1.25	25.45 ± 4.45	33.94 ± 9.53	48.10 ± 9.15 *	4.16 ± 1.01 *
**tumor-to-organ ratio**					
stomach	3.08 ± 1.07	3.66 ± 0.64	4.69 ± 1.19	11.46 ± 1.82 *	4.33 ± 0.56
kidney	6.11 ± 0.64	6.04 ± 0.87	1.62 ± 0.15 *	7.55 ± 0.48 *	0.96 ± 0.20 *
blood	572 ± 243	398.77 ± 89.59	175.77 ± 31.88 *	280.85 ± 88.51	69.87 ± 38.31 *

* statistically significant (*p* ≤ 0.05) when compared to [^111^In]In-DOTA-MGS5.

**Table 5:** Biodistribution and selected tumor-to-organ ratios of [^111^In]In- (4 h p.i.), [^68^Ga]Ga- (1 h p.i.) and [^177^Lu]Lu-labeled DOTA-[(*N*-Me)1Nal^8^]MGS5 (4 h p.i.) as well as DOTA-MGS5 [19] in A431-CCK2R/A431-mock xenografted BALB/c nude mice. All biodistribution values are reported as percentage of injected activity per gram tissue (% IA/g); mean ± SD (*n* = 4; except [^177^Lu]Lu- DOTA-MGS5 *n* = 5).

	DOTA-[(*N*-Me)1Nal^8^]MGS5 Radiolabeled with			DOTA-MGS5 Radiolabeled with		
**% IA/g**	**indium-111**	**gallium-68**	**lutetium-177**	**indium-111**	**gallium-68**	**lutetium-177**
blood	0.19 ± 0.07 *	2.82 ± 0.88	0.13 ± 0.04	0.05 ± 0.03	1.47 ± 0.82	0.11 ± 0.11
lung	0.21 ± 0.07 *	1.61 ± 0.42	0.20 ± 0.04	0.09 ± 0.05	1.35 ± 0.64	0.17 ± 0.14
heart	0.14 ± 0.03 *	0.95 ± 0.32	0.15 ± 0.02	0.05 ± 0.02	0.82 ± 0.51	0.10 ± 0.06
muscle	0.12 ± 0.05	0.45 ± 0.08	0.15 ± 0.06	0.26 ± 0.28	0.22 ± 0.17	0.10 ± 0.02
spleen	0.32 ± 0.05 *	1.19 ± 0.25	0.27 ± 0.04	0.16 ± 0.04	0.68 ± 0.38	0.27 ± 0.18
intestine	0.81 ± 0.10	0.82 ± 0.18	1.47 ± 0.26	1.15 ± 0.31	0.79 ± 0.35	1.02 ± 0.23
liver	1.39 ± 0.16 *	2.25 ± 0.55 *	1.05 ± 0.15	0.46 ± 0.03	1.02 ± 0.52	1.02 ± 0.80
kidneys	6.33 ± 0.89 *	7.70 ± 3.10	4.69 ± 0.68	3.88 ± 0.45	5.71 ± 1.38	3.45 ± 0.91
pancreas	1.37 ± 0.22	0.87 ± 0.29 *	0.60 ± 0.16 *	1.6. ± 0.50	1.83 ± 0.42	1.91 ± 0.91
stomach	4.20 ± 0.51 *	1.63 ± 0.41 *	2.37 ± 0.33	8.24 ± 2.40	5.12 ± 1.13	6.26 ± 4.28
A431-mock xenograft	0.38 ± 0.08	1.51 ± 0.98	0.28 ± 0.04 *	0.3 ± 0.12	0.78 ± 0.26	0.19 ± 0.03
A431-CCK2R xenograft	48.10 ± 9.15 *	15.67 ± 2.21 *	35.13 ± 6.32 *	23.49 ± 1.25	23.25 ± 4.70	22.89 ± 4.67
**tumor-to-organ ratio**	**indium-111**	**gallium-68**	**lutetium-177**	**indium-111**	**gallium-68**	**lutetium-177**
stomach	11.5 ± 1.82 *	10.2 ± 3.50 *	14.9 ± 2.63 *	3.08 ± 1.07	4.56 ± 0.80	4.76 ± 2.62
kidney	7.55 ± 0.48 *	2.36 ± 1.14 *	7.72 ± 2.26	6.11 ± 0.64	4.10 ± 0.26	6.96 ± 2.23
blood	281 ± 89	6.03 ± 2.26 *	296 ± 141	572 ± 243	18.93 ± 7.51	463 ± 396

* statistically significant (*p* ≤ 0.05) when compared to the DOTA-MGS5 counterpart.

## Data Availability

The data presented in this study are contained within the article and the supplementary material.

## Supplementary Materials

The following supporting information can be downloaded at: https://www.mdpi.com/article/10.3390/ph16020278/s1, Figure S1: UV-trace and respective MALDI-TOF spectrum of (a) DOTA-MGS5 (b) DOTA-[2Nal^8^]MGS5, (c) DOTA-[DLys^1^]MGS5, (d) DOTA-[(*N*-Me)1Nal^8^]MGS5, (e) DOTA-MGS5[NHCH_3_] and (f) DOTA-[Phe^8^]MGS5, Figure S2 Radiochromatogram of (a) [^111^In]In-DOTA-MGS5 (b) [^111^In]In-DOTA-[2Nal^8^]MGS5, (c) [^111^In]In-DOTA-[DLys^1^]MGS5, (d) [^111^In]In-DOTA-MGS5[NHCH_3_] and (e) [^111^In]In-DOTA-[Phe^8^]MGS5, Figure S3 Radiochromatogram of (a) [^111^In]In-DOTA-[(*N*-Me)1Nal^8^]MGS5 (b) [^68^Ga]Ga-DOTA-[(*N*-Me)1Nal^8^]MGS5 and (c) [^177^Lu]Lu-DOTA-[(*N*-Me)1Nal^8^]MGS5, Table S1 Summary of the analytical data of DOTA-[(N-Me)1Nal^8^]MGS5 labeled with different radiometals, Figure S4 Cell internalization of DOTA-[(*N*-Me)1Nal^8^]MGS5 labeled with different radiometals 2 h after incubation. Gray: indium-111, striped: lutetium-177 and checked: gallium-68. Figure S5 Representative radiochromatograms of the ^111^In-labeled peptides post preparation (p.p.), as well as kidney and liver samples obtained from BALB/c mice 10 min p.i. Pink: DOTA-[2Nal^8^]MGS5, green: DOTA-[DLys^1^]MGS5, purple: DOTA-[(*N*-Me)1Nal^8^]MGS5, blue: DOTA-MGS5[NHCH_3_].
