# Supplementary material for: Effects of Side Chain and Peptide Bond Modifications on the Targeting Properties of Stabilized Minigastrin Analogs
Source: Pharmaceuticals (Basel). 2023 Feb 13;16(2):278. doi: 10.3390/ph16020278 (PMC9959130; doi:10.3390/ph16020278)
Supplement: Supplementary file 1 [file pharmaceuticals-16-00278-s001.zip › pharmaceuticals-2144055-supplementary.pdf]

Table of content

Figure S1. UV-trace and respective MALDI-TOF spectrum of (a) DOTA-MGS5 (b) DOTA-[2Nal^{8}]MGS5, (c) DOTA-[DLys^{1}]MGS5, (d) DOTA-[(*N*-Me)1Nal^{8}]MGS5, (e) DOTA-MGS5[$NHCH_3$] and (f) DOTA-[Phe^{8}]MGS5. 3

Figure S2. Radiochromatogram of (a) [^{111}In]In-DOTA-MGS5 (b) [^{111}In]In-DOTA-[2Nal^{8}]MGS5, (c) [^{111}In]In-DOTA-[DLys^{1}]MGS5, (d) [^{111}In]In-DOTA-MGS5[$NHCH_3$] and (e) [^{111}In]In-DOTA-[Phe^{8}]MGS5. 4

Figure S3. Radiochromatogram of (a) [^{111}In]In-DOTA-[(*N*-Me)1Nal^{8}]MGS5 (b) [^{68}Ga]Ga-DOTA-[(*N*-Me)1Nal^{8}]MGS5 and (c) [^{177}Lu]Lu-DOTA-[(*N*-Me)1Nal^{8}]MGS5. 5

Table S1. Summary of the analytical data of DOTA-[(*N*-Me)1Nal^{8}]MGS5 labeled with different radiometals 6

Figure S4. Cell internalization of DOTA-[(*N*-Me)1Nal^{8}]MGS5 labeled with different radiometals 2 h after incubation. Gray: indium-111, striped: lutetium-177 and checked: gallium-68. 6

Figure S5. Representative radiochromatograms of the ^{111}In-labeled peptides post preparation (p.p.), as well as kidney and liver samples obtained from BALB/c mice 10 min p.i. Pink: DOTA-[2Nal^{8}]MGS5, green: DOTA-[DLys^{1}]MGS5, purple: DOTA-[(*N*-Me)1Nal^{8}]MGS5, blue: DOTA-MGS5[$NHCH_3$]. 7

(a)

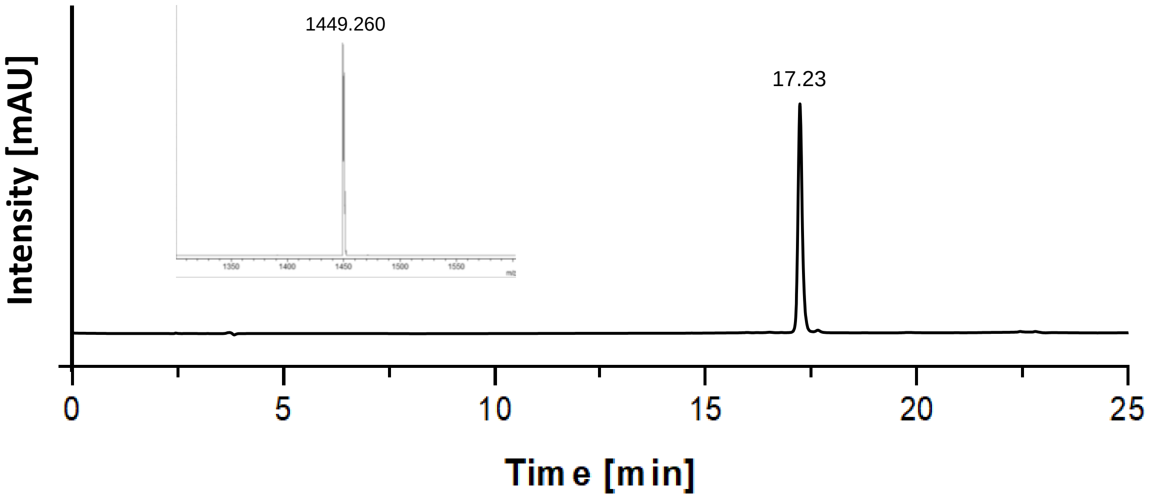
1449.260
17.23
Intensity [mAU]
0
5
10
15
20
25
Tim e [m in]

(b)

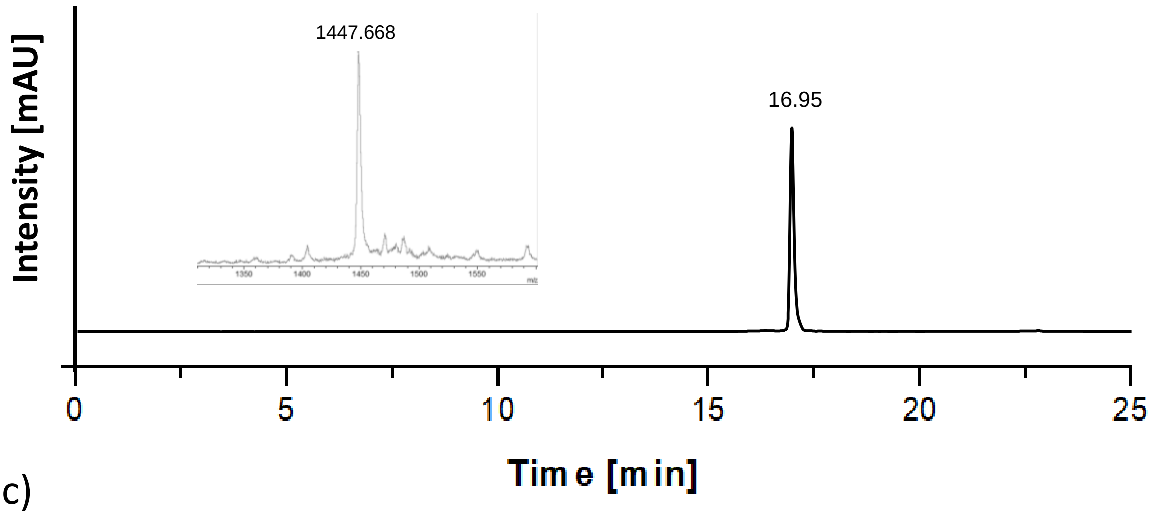
1447.668
16.95
Intensity [mAU]
0
5
10
15
20
25
Tim e [m in]

(c)

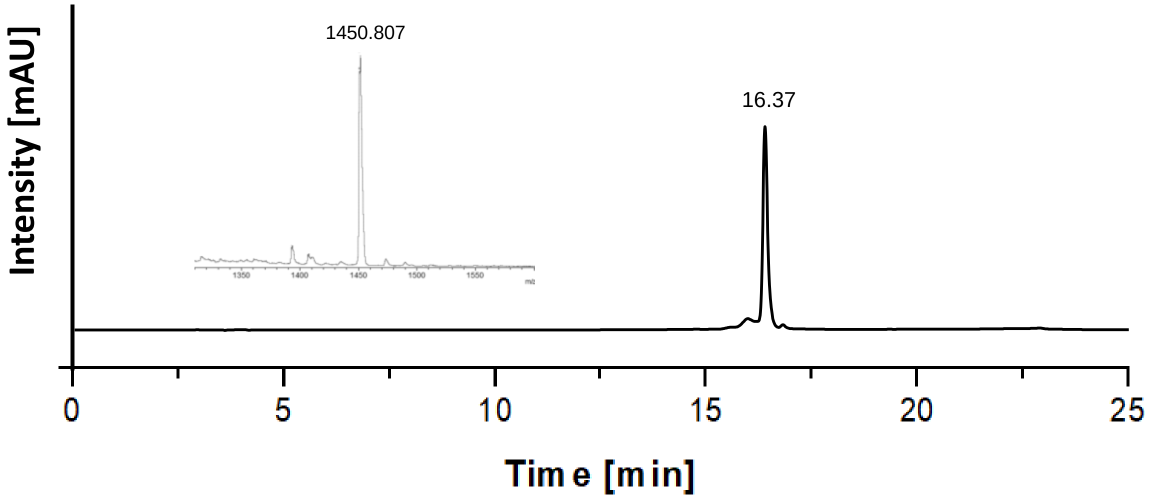
1450.807
16.37
Intensity [mAU]
0
5
10
15
20
25
Tim e [m in]

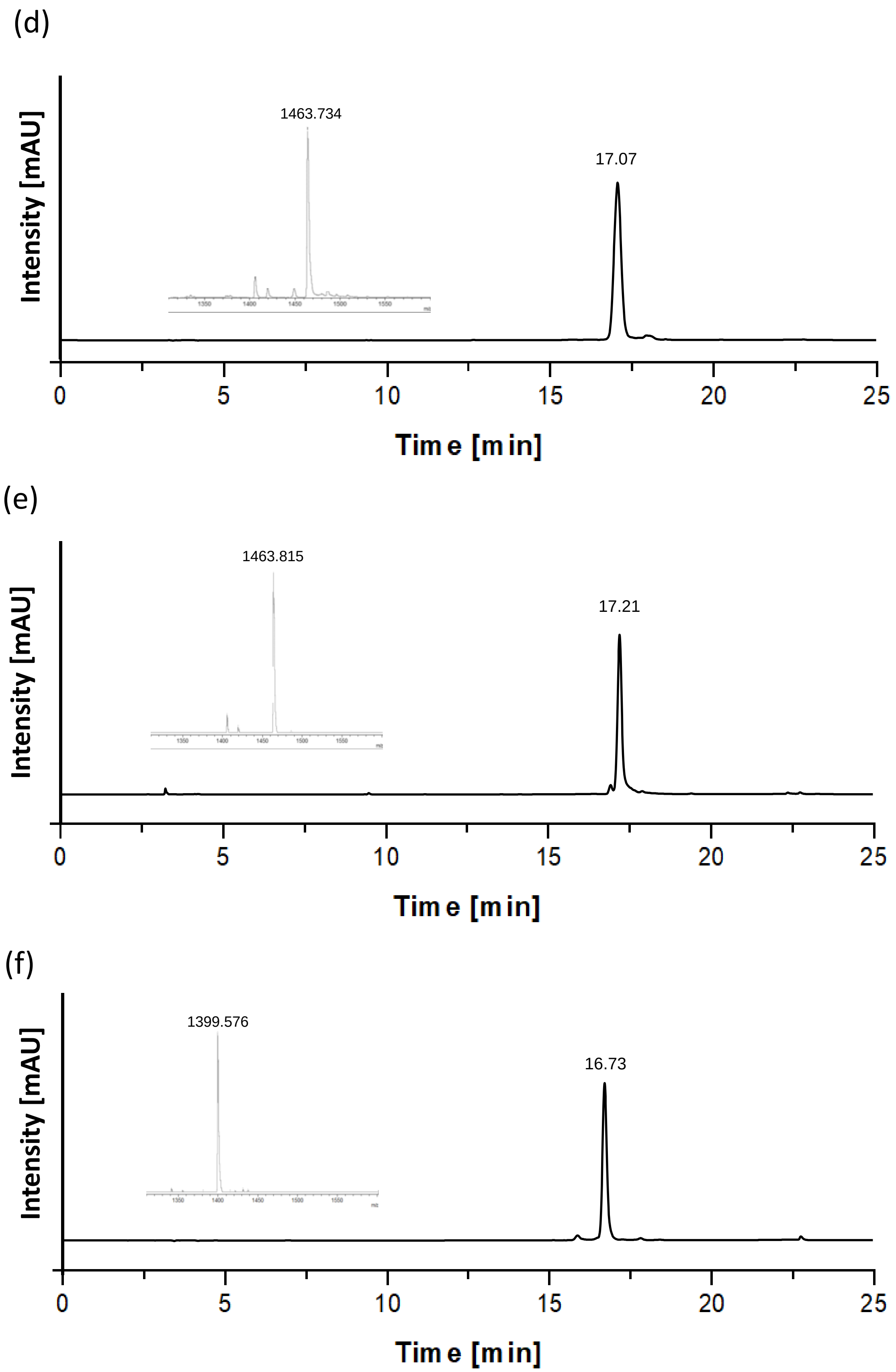

Figure S1. UV-trace and respective MALDI-TOF spectrum of (**a**) DOTA-MGS5 (**b**) DOTA-[2Nal^{8}]MGS5, (**c**) DOTA-[DLys^{1}]MGS5, (**d**) DOTA-[(*N*-Me)1Nal^{8}]MGS5, (**e**) DOTA-MGS5[$NHCH_3$] and (**f**) DOTA-[Phe^{8}]MGS5.

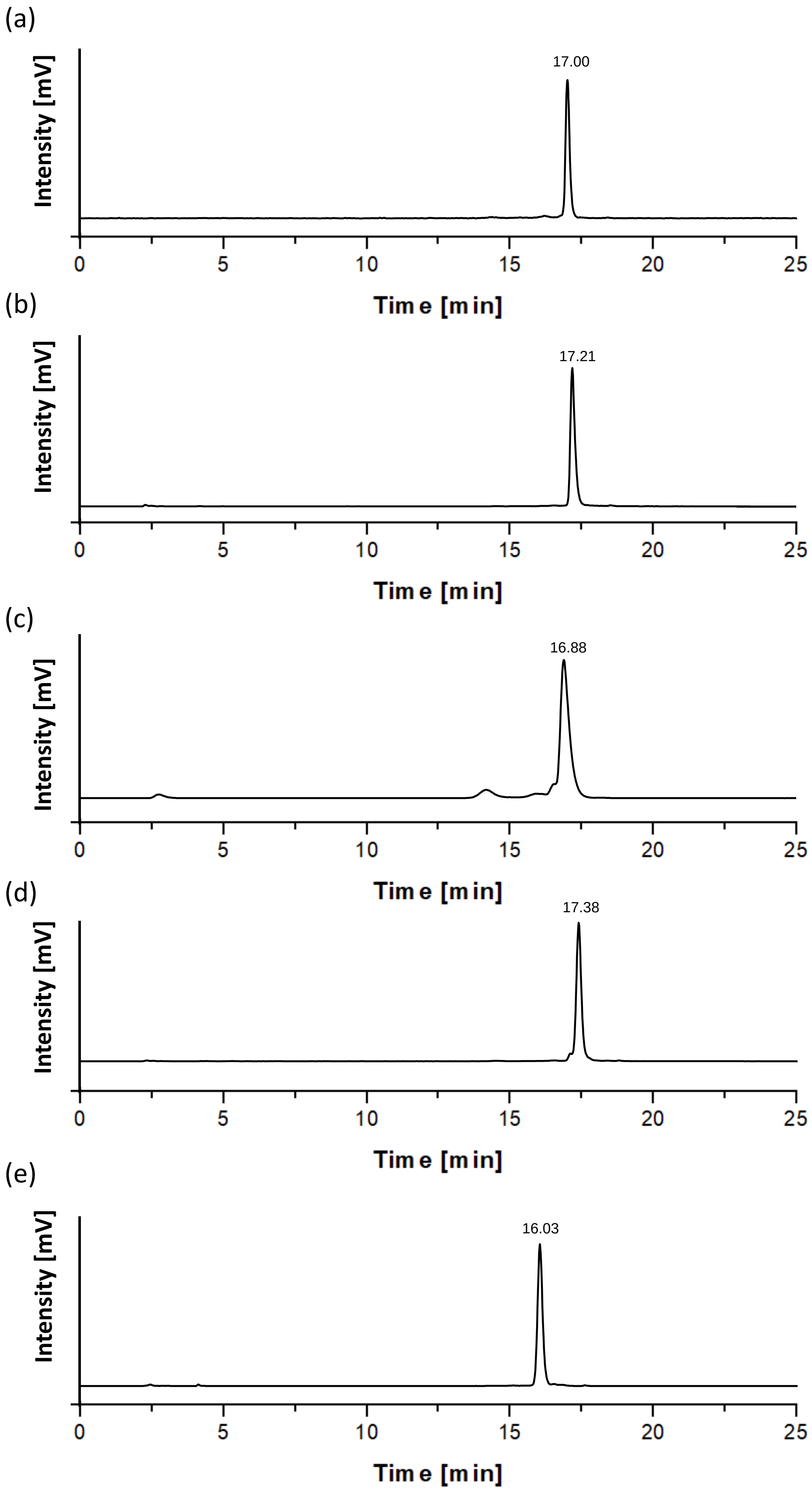

Figure S2. Radiochromatogram of (**a**) $[^{111}In]$In-DOTA-MGS5 (**b**) $[^{111}In]$In-DOTA-$[2Nal^{8}]$MGS5, (**c**) $[^{111}In]$In-DOTA-$[DLys^{1}]$MGS5, (**d**) $[^{111}In]$In-DOTA-MGS5$[NHCH_3]$ and (**e**) $[^{111}In]$In-DOTA-$[Phe^{8}]$MGS5.

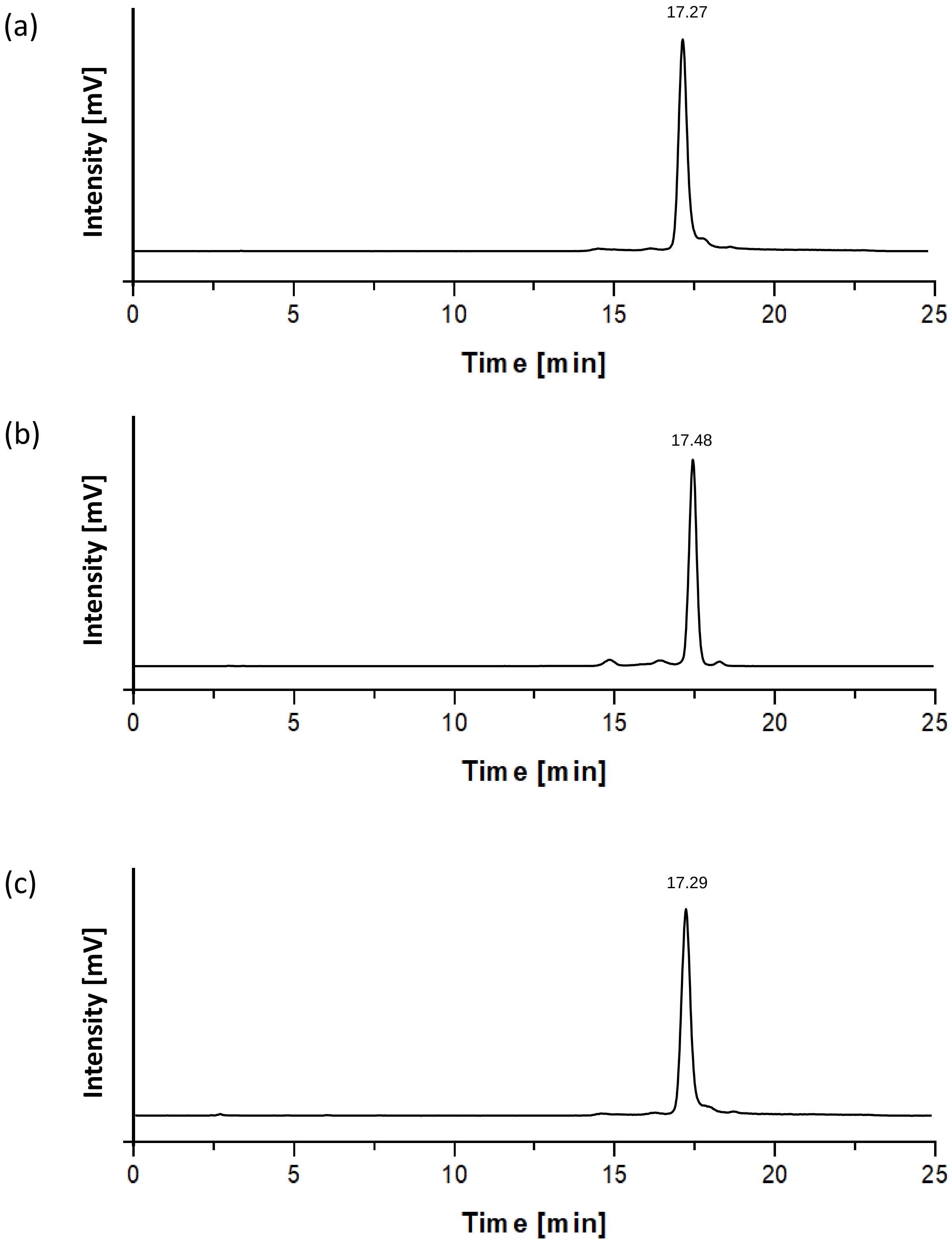

Figure S3. Radiochromatogram of (**a**) [^{111}In]In-DOTA-[(*N*-Me)1Nal^{8}]MGS5 (**b**) [^{68}Ga]Ga-DOTA-[(*N*-Me)1Nal^{8}]MGS5 and (**c**) [^{177}Lu]Lu-DOTA-[(*N*-Me)1Nal^{8}]MGS5.

In vitro Characterization of DOTA-[(N-Me)1Nal^{8}]MGS5 Labeled with Different Radiometals

Labeling with different radiometals of DOTA-[(*N*-Me)1Nal^{8}]MGS5 resulted in a similar hydrophilicity of DOTA-[(*N-Me*)1Nal^{8}]MGS5 labeled with different radiometals with values of −2.4 ± 0.3 for indium−111, −2.0 ± 0.1 for lutetium−177 and −2.2 ± 0.1 for gallium−68. Only minor differences in binding to serum proteins were observed depending on the radiometal used for labeling. For the time point of 4 h after incubation, the highest protein binding was found for [^{111}In]In-DOTA-[Phe^{8}]MGS5 (31.9 ± 0.3%), followed by [^{68}Ga]Ga-DOTA-[(*N*-Me)1Nal^{8}]MGS5 (25.5 ± 1.5%) and [^{177}Lu]Lu-DOTA-[(*N*-Me)1Nal^{8}]MGS5 (25.4 ± 2.3%). Similar values were found also for ^{177}Lu-labeled (28.7 ± 7.3%) and ^{111}In-labeled peptide (39.8 ± 4.2%) DOTA-[(*N*-Me)1Nal^{8}]MGS5 for the time point of 24 h after incubation. Incubation in fresh human serum further confirmed a very high stability in vitro of DOTA-[(*N*-Me)1Nal^{8}]MGS5 labeled with different radiometals. No free radiometal and only minor amounts of peptide related radiometabolites could be monitored in the radiochromatograms at 4 h after incubation. Also at the later time point of 24 h after incubation, ≥ 98% intact radiopeptide was observed for [^{111}In]In-DOTA-[2Nal^{8}]MGS5 and [^{177}Lu]Lu-DOTA-[2Nal^{8}]MGS5. The different results of the in vitro characterization of DOTA-[(*N*-Me)1Nal^{8}]MGS5 labeled with different radiometals are summarized in Table 1. As a control, the stability in PBS (n = 1) was also tested, confirming a high stability for the DOTA-complex with indium−111 (96.2%), gallium−68 (99.2%) and lutetium−177 (99%) for 4 h after incubation.

Table S1. Summary of the analytical data of DOTA-[(*N*-Me)1Nal^{8}]MGS5 labeled with different radiometals

**Compound**	**Binding to Serum Proteins [%]** n = 2		**LogD** n = 8	**Intact Radiopeptide in Human Serum [%]** n = 2	
	**4 h**	**24 h**		**4 h**	**24 h**
[^{111}In]In-DOTA-[(*N*-Me)1Nal^{8}]MGS5	31.9 ± 0.3	39.8 ± 4.2	−2.4 ± 0.3	99.3 ± 0.9	99.6 ± 0.1
[^{177}Lu]Lu-DOTA-[(*N*-Me)1Nal^{8}]MGS5	25.4 ± 2.3	28.7 ± 7.3	−2.0 ± 0.1	98.3 ± 0.5	98.5 ± 1.2
[^{68}Ga]Ga-DOTA-[(*N*-Me)1Nal^{8}]MGS5	25.5 ± 1.5	n.d.	−2.2 ± 0.1	99.9 ± 0.0	n.d.

Cell Internalization of DOTA-[(N-Me)1Nal^{8}]MGS5 Labeled with Different Radiometals

The receptor-specific internalization of DOTA-[(*N*-Me)1Nal^{8}]MGS5 labeled with different radiometals was studied for the time point of 2 h after incubation. In analogy to the varying tumor uptake observed in vivo, [^{111}In]In-DOTA-[(*N*-Me)1Nal^{8}]MGS5 demonstrated the highest receptor-mediated cell internalization with the value of 31.5 ± 6.7%. [^{177}Lu]Lu-DOTA-[(*N*-Me)1Nal^{8}]MGS5 showed a similar receptor-specific cell uptake of 30.8 ± 5.5%. For [^{68}Ga]Ga-DOTA-[(*N*-Me)1Nal^{8}]MGS5 a much lower cell uptake of 20.4 ± 2.3% was found for the same time point.

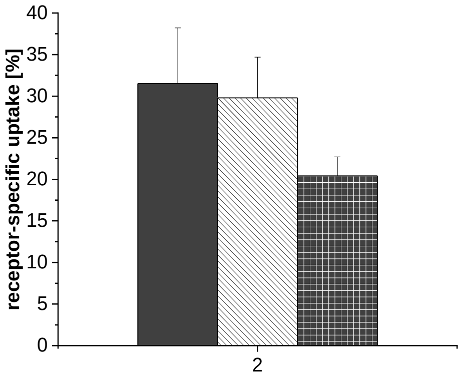

Figure S4. Cell internalization of DOTA-[(*N*-Me)1Nal^{8}]MGS5 labeled with different radiometals 2 h after incubation. Gray: indium−111, striped: lutetium−177 and checked: gallium−68.

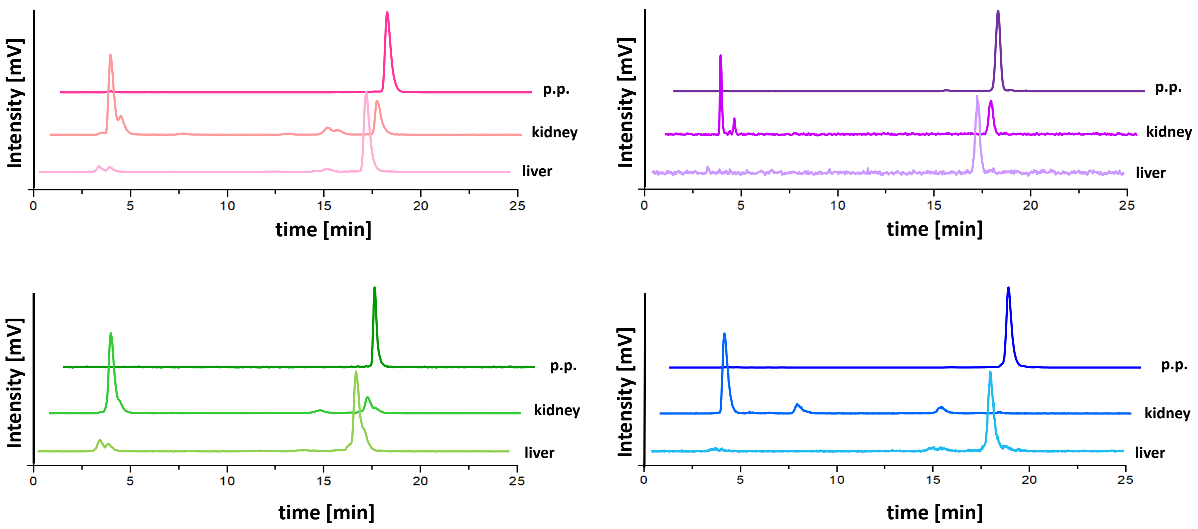

Figure S5. Representative radiochromatograms of the ^{111}In-labeled peptides post preparation (p.p.), as well as kidney and liver samples obtained from BALB/c mice 10 min p.i. Pink: DOTA-[2Nal^8]MGS5, green: DOTA-[DLys^1]MGS5, purple: DOTA-[(*N*-Me)1Nal^8]MGS5, blue: DOTA-MGS5[NHCH$_3$].
